# Performance of a two-item sleep quality measure (PSQI-2): a comprehensive evaluation in a multiethnic cohort (MESA Study)

**DOI:** 10.21203/rs.3.rs-8413149/v1

**Published:** 2026-02-03

**Authors:** Luiz Menezes-Júnior

**Affiliations:** Federal University of Ouro Preto

**Keywords:** Sleep quality, PSQI, validation, epidemiological methods, sleep disorders

## Abstract

**Objective:**

To evaluated the abbreviated two-item Pittsburgh Sleep Quality Index (PSQI-2) against the full PSQI in a multi-ethnic cohort.

**Methods:**

We analyzed data from 2,237 participants from the MESA Sleep Ancillary Study. The full PSQI was adapted by integrating actigraphy data for sleep duration, latency, and efficiency components, while maintaining the original seven-component structure scored 0–3. The PSQI-2 was derived from two components: sleep duration (questionnaire-based) and subjective sleep quality. Validation analyses included correlation analysis; ROC curves for three PSQI cutpoints (> 5, > 7, >10) with sensitivity/specificity calculations, Bland-Altman analysis for agreement, bootstrap internal validation, and logistic regression for demographic, clinical, and sleep-related covariates.

**Results:**

Poor sleep quality was prevalent (65.6% by PSQI > 5; 65.7% by PSQI-2 > 1). The PSQI-2 showed strong correlation with the full PSQI (r = 0.520, p < 0.001), consistent across gender and age subgroups. Both measures identified similar risk patterns: Black and Hispanic participants had higher odds of poor sleep, and obesity, sleep disorders, daytime sleepiness, and evening chronotype consistently increased poor sleep odds. The PSQI-2 demonstrated good discriminant validity across PSQI cutpoints (AUC: 0.785 for > 5, 0.748 for > 7, 0.750 for > 10), with sensitivity ranging from 71.8–86.8% and specificity from 46.3–79.1%.

**Conclusion:**

The PSQI-2 shows strong validity and consistent performance with the full PSQI, effectively identifying poor sleep quality and associated factors. Its brevity makes it suitable for large-scale studies and clinical screening.

## Background

Sleep quality is a fundamental pillar of human health, and its impairment is a significant public health concern associated with a wide range of adverse outcomes, including non-communicable chronic diseases, metabolic disorders, and cognitive decline [1, 2]. The Pittsburgh Sleep Quality Index (PSQI), developed by Buysse et al. (1989), has been established as a gold-standard self-report instrument for evaluating sleep quality in both clinical and research settings [3]. Its reliability and validity have been examined in diverse populations [4–6], confirming its utility while also revealing that its psychometric properties can be optimized in specific groups [7].

Despite its widespread use, the comprehensive nature of the 19-item PSQI presents practical limitations. In large-scale epidemiological studies that simultaneously investigate multiple health domains, extensive questionnaires can lead to respondent fatigue, increased missing data, and ultimately, constraints on the breadth of research [8, 9]. This challenge has spurred the development and validation of abbreviated instruments, such as the two-item PSQI (PSQI-2), which focuses on the core dimensions of sleep duration and subjective sleep quality [10, 11]. In a population-based household survey in Brazil, psychometric analyses supported a two-factor structure based on PSQI items, with excellent internal consistency, clear gradients in poor sleep prevalence across score levels, and good concurrent and known-group validity [10]. More recently, validation in the MrOS Sleep Study extended this evidence to a longitudinal context among community-dwelling older men, showing strong agreement with the full PSQI, excellent discriminatory accuracy, moderate test–retest reliability, and good responsiveness to clinically meaningful changes in sleep over time [11]. Together, these findings support the PSQI-2 as a valid, reliable, and pragmatic alternative for sleep quality assessment in large-scale epidemiological and longitudinal studies.

Therefore, the brevity of such tools makes them particularly suitable for large studies where time and questionnaire space are limited. However, the performance of these short forms, especially in diverse, multi-ethnic populations and against objective sleep measures, requires further robust characterization [12]. The Multi-Ethnic Study of Atherosclerosis (MESA) Sleep Ancillary Study provides an ideal platform to address this research gap. MESA itself is a landmark, prospective cohort study initiated by the National Heart, Lung, and Blood Institute (NHLBI) in 1999–2000 to investigate the prevalence, correlates, and progression of subclinical cardiovascular disease in a sex-balanced, multi-ethnic cohort [13]. The MESA Sleep Ancillary Study augmented this rich dataset with a comprehensive sleep assessment protocol, including 7-day actigraphy, in addition to self-report questionnaires [14]. This unique combination of subjective and objective sleep data within a large, community-dwelling, multi-ethnic population offers an unparalleled opportunity to validate an abbreviated sleep instrument against a robust criterion standard.

Therefore, this study aims to validate an adapted version of the PSQI-2 within the MESA Sleep study. We will examine its psychometric properties, diagnostic accuracy against the full PSQI, and its relationship with key demographic, clinical, and objective sleep measures.

## Methods

### Study population and design

The Multi-Ethnic Study of Atherosclerosis (MESA) is a prospective cohort study sponsored by the National Heart, Lung, and Blood Institute (NHLBI). Its primary goal is to investigate risk factors for the development and progression of subclinical cardiovascular disease in a diverse population [13]. The datasets analyzed for this study were provided by the National Sleep Research Resource (Sleep Data, https://sleepdata.org) [15].

The study began in 1999–2000, enrolling 6,814 participants aged 45–84 years who were free of clinically diagnosed cardiovascular disease at baseline. Participants were recruited from six field centers across the United States: Baltimore, MD; Chicago, IL; Los Angeles, CA; New York, NY; Saint Paul, MN; and Winston-Salem, NC. A key strength of MESA is its deliberate inclusion of a multi-ethnic population, with the cohort comprising White, Black, Hispanic, and Chinese-American individuals. Participants have undergone serial clinical examinations, with the most recent (the seventh exam) being conducted from 2022 to 2024. Between exams, annual follow-up contacts are conducted to assess clinical cardiovascular events and other health outcomes [13].

The MESA Sleep Ancillary Study was conducted to examine the relationships between sleep characteristics and cardiovascular disease risk. Data collection for this ancillary study occurred in close temporal proximity to the MESA Exam 5 (2010–2013) [14]. Out of the 4,077 participants who attended Exam 5, 2,261 individuals were enrolled in the MESA Sleep Ancillary Study and provided objective and subjective sleep data. Our present analysis focuses on the subset of these participants who completed the relevant sleep questionnaires and objective measurements.

A major strength of the MESA Sleep study is its multi-method assessment of sleep, which includes for this study: Actigraphy, with participants wore an Actiwatch Spectrum (Philips Respironics) on the non-dominant wrist for 7 consecutive days to objectively estimate habitual sleep patterns, including sleep duration, sleep efficiency, and night-to-night variability in sleep timing, in their home environment [14]. Furthermore, participants completed self-report questionnaires, including the Women’s Health Initiative Insomnia Rating Scale (WHIIRS), Epworth Sleepiness Scale (ESS), Modified Horne-Ostberg Morningness-Eveningness Questionnaire (MEQ) and other instruments assessing sleep disorder screening, captures self-reported physician diagnoses of specific sleep disorders.

## Variables

### Sleep quality

#### Full sleep quality questionnaire

The standard PSQI was not administered in the MESA Sleep Ancillary Study, therefore, an adapted measure of full sleep quality was constructed. This adaptation leveraged core sleep domains assessed by the study’s existing validated questionnaires. Thus, the questionnaire was developed to mirror the structure and scoring of the original instrument while also leveraging the unique strengths of the dataset, which included both self-reported questionnaire data and objective actigraphy measures. The primary goal was to create a composite sleep quality score that integrated subjective perceptions with behavioral sleep patterns.

The full PSQI was constructed to comprise the same seven components as the original PSQI, each scored on a 0–3 scale, where 0 indicates no difficulty and 3 indicates severe difficulty.

Subjective sleep quality was directly derived from the question assessing overall typical night’s sleep (typicalslp5). The original 5-point scale was recoded to the PSQI’s 4-point scale (0–3), consolidating the two poorest categories into a single top score. Sleep latency was calculated by integrating both questionnaire and actigraphy data to capture the multifaceted nature of sleep onset difficulty. Initially, a weighted average of weekly sleep onset latency was calculated from weekday (avgonsetlatencywd5) and weekend (avgonsetlatencywe5) data. This continuous measure (in minutes) was then categorized into PSQI scoring bands. Furthermore, the frequency of self-reported trouble falling asleep (trbleslpng5) was scored on a 0–3 scale. The final sleep latency component score was generated by summing the actigraphy and questionnaire scores and recategorizing the combined value.

For sleep duration, objective actigraphy data was prioritized for this component to reflect actual sleep time rather than time in bed. Therefore, a weighted average of weekly total sleep time was calculated from weekday (avgmainsleepwd5) and weekend (avgmainsleepwe5) data. This value was then scored based on the established PSQI criteria. Sleep efficiency, defined as the ratio of total sleep time to total time in bed multiplied by 100, was directly obtained from the actigraphy-derived variable (slp_eff5). This objective measure was then scored using the standard PSQI thresholds.

Sleep disturbances was constructed from the frequency of six specific sleep problems reported in the questionnaire: trouble falling asleep (trbleslpng5); waking up in the middle of the night (wakeup5); waking up too early (wakeearly5); trouble getting back to sleep after waking up (bcksleep5); snoring (snored5); and stopping breathing during sleep (stpbrthng5).

Each disturbance was scored on a 0–3 scale based on its frequency. The sum of these six scores was calculated, and since the adapted scale had a lower maximum (18) than the original PSQI (27), the scoring thresholds were proportionally adjusted to maintain a 0–3 component score.

Use of sleep medication, was evaluated with the frequency of sleeping pill use (slpngpills5) was directly scored on the 0–3 scale. Finally, the daytime dysfunction and sleepiness, was evaluated by combining daytime sleepiness, with the frequency of feeling overly sleepy during the day (sleepy5); and frequency of sleep difficulties causing irritability (irritable5).

Each was scored on a 0–3 scale. The sum of these two scores was then recategorized to generate the final component score, ensuring it remained on the standard 0–3 scale.

The global full PSQI score was computed as the sum of the seven component scores, with a higher score indicating worse sleep quality. The traditional cut-off of > 5 was used to classify participants as having “poor” sleep quality.

#### PSQI-2

The PSQI-2 development followed the conceptual framework proposed by Menezes-Júnior et al. (2025). The two-component structure comprised sleep duration (identical to component 1 of the full adapted PSQI) and subjective sleep quality (derived from typicalslp5, identical to component 4 of the full PSQI). This approach aligns with the theoretical foundation that these two domains capture the essential elements of sleep quality assessment. The PSQI-2 score ranged from 0–6, with the established cutoff of ≥ 2 indicating poor sleep quality, consistent with validation studies in other populations [10]

#### Coviariates

The analysis adjusted for a comprehensive set of covariates known or suspected to be associated with sleep quality and health outcomes. Sociodemographic characteristics included sex (female, male), age group (54–64 years, 65–74 years, 75 + years), and self-reported race/ethnicity (White, Black, Hispanic, Chinese-American). Employment status was detailed by assessing whether participants were currently working and, if so, their predominant work shift (daytime, night/rotating, irregular), with a distinct category for those not in the workforce. Behavioral and health-related factors accounted for were current smoking status (yes, no), categorized further into never, former, or current smoker, and body mass index (BMI) categorized as normal, overweight, or obese. Additional behavioral covariates included the habit of taking regular naps (yes, no) and the use of a CPAP or BiPAP machine for sleep-disordered breathing (yes, no). Furthermore, the model adjusted for several key sleep-related conditions and traits, namely the presence of clinically significant insomnia, restless legs syndrome, a prior diagnosis of sleep apnea, and excessive daytime sleepiness. Finally, to account for individual differences in circadian preference, chronotype was also included as a covariate, classified as morning, intermediate, or evening type.

#### Statistical analysis

Our validation approach employed comprehensive psychometric analyses following established guidelines for instrument validation. All analyses were conducted using Stata version 17 (StataCorp, College Station, TX).

The analysis proceeded in two sequential phases. The first phase focused on descriptive characterization, data preparation, and internal consistency assessment. We examined distributional properties of both PSQI instruments using histograms and formal normality testing (Shapiro-Wilk tests). Prevalence estimates of poor sleep quality according to various definitions provided context for the clinical relevance of findings. Component-level analyses included examination of the distribution and intercorrelation of PSQI-2 components, assessing the fundamental structure of the abbreviated instrument. Internal consistency reliability was evaluated using Cronbach’s alpha and McDonald’s omega coefficients for the full PSQI, with values ≥ 0.70 considered indicative of acceptable reliability.

The second phase encompassed psychometric validation. Concurrent validity assessment included Pearson correlations between full PSQI and PSQI-2 scores, with supplementary linear regression modeling the functional relationship between instruments. Scatter plots with regression lines provided visual representation of this relationship. Agreement between instruments was quantified using Bland-Altman analysis with regression-based rescaling of the PSQI-2 to the PSQI metric, calculating mean bias, standard deviation of differences, and 95% limits of agreement. This approach acknowledges the different scaling of the two instruments while enabling direct comparison.

Diagnostic performance evaluation employed receiver operating characteristic (ROC) analysis using the full PSQI cutoffs (> 5, > 7, >10) using logistic regression. The area under the receiver operating characteristic curve (AUC) was calculated with cluster bootstrapping to derive confidence intervals. Recognizing the importance of instrument performance across demographic groups, we conducted stratified analyses by gender and age (using median split).

## Results

### 3.1 Sample characteristics

Table 1 presents the prevalence and 95% confidence intervals (CI) for sociodemographic, health, and sleep-related characteristics of the MESA Sleep cohort (n = 2,237), stratified by sleep quality according to the PSQI (> 5) and PSQI-2 (> 1) thresholds. The sample was composed predominantly of women (53.6%), participants aged 54–64 years (36.2%), and racially diverse groups including 37.1% White, 27.5% Black, 23.5% Hispanic, and 11.9% Chinese-American. Obesity affected 35.5% of the sample, and 6.7% were current smokers. Sleep disorders were frequently self-reported: 9.0% had a diagnosis of sleep apnea, 6.6% insomnia, and 4.7% restless legs syndrome. Excessive daytime sleepiness was reported by 13.8%, and 7.4% identified as having an evening chronotype. Approximately 59.9% of participants reported regular napping (Table 1).

### 3.2 Sleep quality

**Figure 1:**
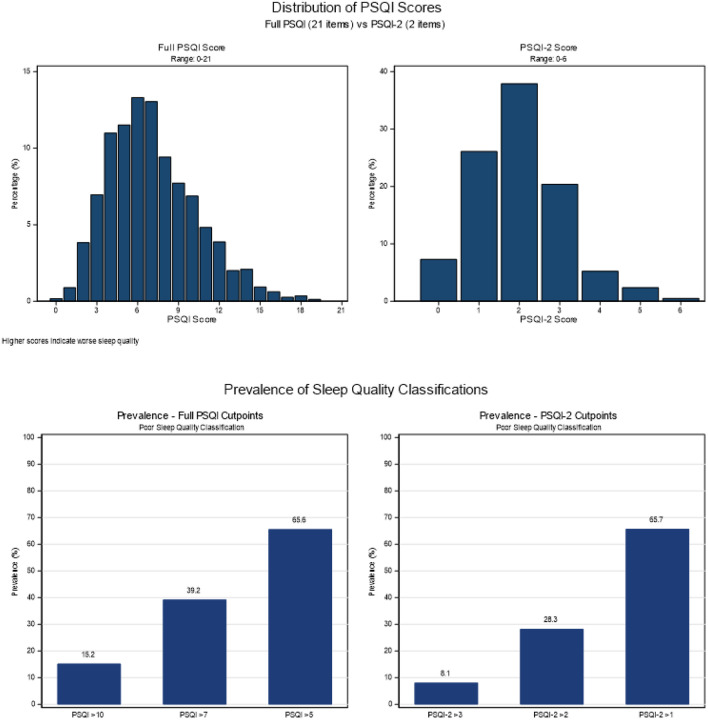
Distribution of PSQI and PSQI-2 scores, and prevalence of poor sleep quality according PSQI scale and cutoff values in the MESA Sleep cohort.

**Figure 2:**
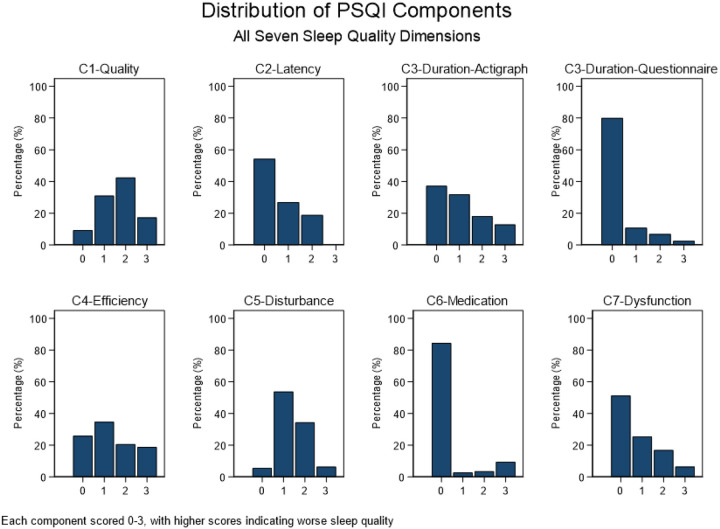
Distribution of PSQI components in the MESA Sleep cohort.

Distributions of PSQI components and total scores are illustrated in Figs. 1–2. The mean full PSQI score was 7.1 (SD = 3.2, range: 0–19), and the abbreviated PSQI-2 showed a mean score of 2.0 (SD = 1.1, range: 0–6). Overall, 65.6% of participants had poor sleep quality by the PSQI (> 5), while 65.7% met the cutoff for reduced sleep quality by the PSQI-2 (> 1) (Fig. 1). Overall, patterns of association between sociodemographic and health variables were consistent for both PSQI and PSQI-2 classifications (Table 1).

Sleep disturbances were the most prevalent PSQI component (94.5% any impairment, 40.7% moderate-severe), followed by daytime dysfunction (48.7% any, 23.3% moderate-severe) and sleep latency problems (45.8% any, 18.8% moderate-severe). Sleep efficiency impairments affected 39.4% of participants, while 31.0% had problematic sleep duration. Only 23.2% of participants had no impaired components, whereas 21.5% had three or more impaired components. Sleep medication use was least common (15.6% any use, 9.4% regular use) (Fig. 2).

**Figure 3:**
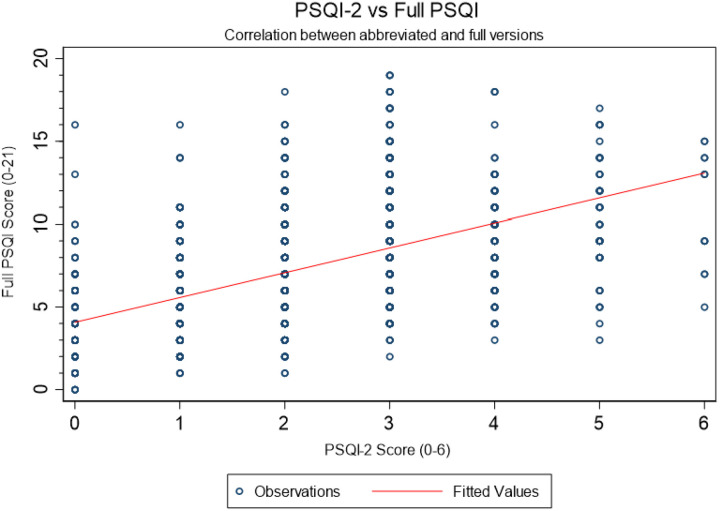
Correlation between PSQI and PSQI-2 total scores in the MESA Sleep cohort. Scatterplot with regression line (r = 0.520, p < 0.001) showing linear association and consistency across score ranges.

The PSQI-2 showed strong correlation with the full PSQI (r = 0.520, p < 0.001) (Fig. 3), which remained consistent across gender (women: r = 0.523; men: r = 0.516) and age groups (≤ 69 years: r = 0.534; >69 years: r = 0.510). Furthermore, logistic regression analyses revealed similar association patterns across PSQI measures. Significant racial disparities existed, with Black and Hispanic participants showing 40–47% higher odds of poor sleep. Obesity, sleep disorders (insomnia, restless legs, apnea), daytime sleepiness, and evening chronotype consistently increased poor sleep odds, with generally stronger effects for the full PSQI. Only age associations diverged between measures (Table 2).

### 3.3 Reliability statistics

Continuous performance metrics assessing the predictive correspondence between PSQI-2 and the full PSQI are displayed in Table 3. The PSQI-2 achieved a Brier Score of 0.185 (95% CI: 0.006–0.188), indicating low overall prediction error. The Mean Absolute Error (MAE = 0.371, 95% CI: 0.019–0.377) and Root Mean Square Error (RMSE = 0.430, 95% CI: 0.075–0.434) also reflected good agreement and stability. The Integrated Discrimination Improvement (IDI = 0.187, 95% CI: 0.002–0.332) supported a high degree of discrimination overlap between the PSQI-2 and full PSQI (Table 3).

**Figure 4:**
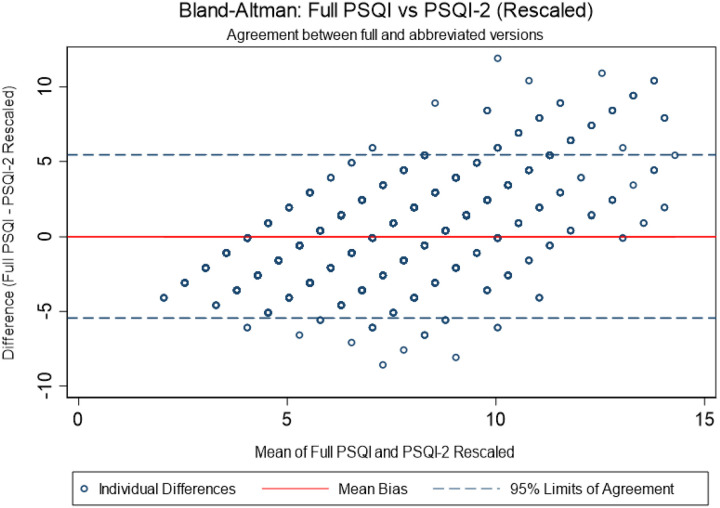
Bland–Altman plot comparing PSQI and PSQI-2 scores in the MESA Sleep cohort. Mean difference close to zero (bias = −0.012), with limits of agreement within ±1.96 SD indicating no systematic bias.

**Figure 5:**
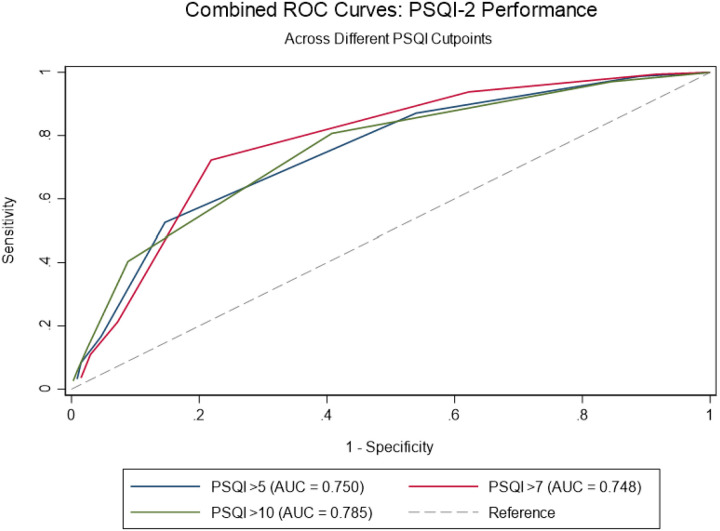
Receiver operating characteristic (ROC) curve for PSQI-2 predicting full PSQI in the MESA Sleep cohort.

A Bland–Altman plot (Fig. 4) demonstrated a negligible mean bias (−0.012) and limits of agreement within ± 1.96 SD, indicating no systematic bias and good concordance across the score range. Furthermore, the ROC curve analysis (Fig. 5) demonstrated that the PSQI-2 showed good discriminant validity for identifying poor sleep quality across different cutpoints of the full PSQI. The area under the curve (AUC) was 0.785 for PSQI > 5, 0.748 for PSQI > 7, and 0.750 for PSQI > 10. At the traditional PSQI > 5 cutpoint, the PSQI-2 > 1 showed sensitivity of 80.4% and specificity of 59.3%, correctly classifying 73.0% of cases. For the PSQI > 7 cutpoint, sensitivity increased to 86.8% while specificity decreased to 46.3%, with 62.1% correct classification. For the most stringent PSQI > 10 cutpoint, the optimal PSQI-2 threshold shifted to ≥ 3, achieving balanced performance with 71.8% sensitivity and 79.1% specificity, correctly classifying 78.0% of cases.

Internal validation by bootstrap resampling (n = 1,000) shows that original Youden Index (0.399 for PSQI-2 > 1) was slightly reduced after bias correction (bootstrap = 0.442), yielding a small bias (0.042) and 95% bias-corrected confidence interval (0.341–0.452) (Table 4).

## Discussion

The present study demonstrates that the abbreviated two-item Pittsburgh Sleep Quality Index (PSQI-2) shows strong validity and consistent performance with the full questionnaire of sleep quality in a large, multi-ethnic cohort of middle-aged and older adults. Overall, the PSQI-2 effectively captured poor sleep quality and reproduced key association patterns observed with the full instrument, supporting its utility as a parsimonious measure in both research and clinical contexts where time and respondent burden are critical considerations.

The strong conceptual alignment between the PSQI-2 and the full PSQI supports the premise that subjective sleep quality and sleep duration represent core dimensions of the broader sleep quality construct. This overlap is reinforced by the consistency of associations observed across both instruments with major sociodemographic, clinical, and behavioral factors. Both measures identified higher likelihood of poor sleep among Black and Hispanic participants, corroborating prior evidence of racial and ethnic disparities in sleep health reported in MESA and other population-based studies [16]. Similarly, expected associations with obesity, diagnosed sleep disorders, daytime sleepiness, and evening chronotype were consistently observed, with slightly stronger effects for the full PSQI, as anticipated given its broader scope [1, 2]. These findings support the construct validity of the PSQI-2 as a concise yet informative indicator of sleep quality.

Our findings align with a growing body of literature supporting the use of abbreviated sleep measures tailored to specific research and clinical needs. Since its original development by Buysse et al. [3, 17] the PSQI has become one of the most widely used subjective sleep instruments worldwide. However, accumulating evidence suggests that the PSQI global score may not be strictly unidimensional, with several studies proposing two- or three-factor structures across different populations [7, 17–21]. These observations provide a strong theoretical basis for the PSQI-2, as a carefully selected subset of items focusing on sleep quality and duration may adequately capture essential aspects of perceived sleep disturbance.

Furthermore, the validation of the PSQI-2 has important implications for both epidemiological research and clinical practice. In large-scale cohort studies such as MESA, where extensive phenotyping must be balanced against feasibility and participant burden, the PSQI-2 provides a pragmatic solution for incorporating sleep quality assessment without substantially increasing survey length or respondent fatigue. This is particularly relevant given the growing body of evidence linking poor sleep quality and short sleep duration to adverse cardiometabolic, cognitive, and mental health outcomes, including hypertension, diabetes, cardiovascular disease, depression, and cognitive decline [22, 23]. The use of brief, validated instruments has been repeatedly emphasized as a key strategy to improve sleep surveillance in population-based studies and public health monitoring.

In clinical settings, the PSQI-2 may function as an efficient first-line screening tool for poor perceived sleep quality, enabling early identification of individuals who may benefit from further diagnostic evaluation or targeted interventions [24, 25]. Its brevity and ease of administration make it especially suitable for primary care, geriatric assessments, and outpatient clinics, where time constraints often limit the use of longer instruments. Prior studies have shown that brief sleep screeners can meaningfully improve detection of sleep problems in routine care and support clinical decision-making without compromising validity [25]. Thus, the PSQI-2 bridges an important gap between comprehensive sleep assessment and real-world clinical and epidemiological feasibility, facilitating broader integration of sleep health into research and practice.

Several limitations should be acknowledged. First, the study relied on subjective sleep measures, which may not fully align with objective sleep parameters, although subjective perception remains clinically meaningful. The MESA study collected extensive objective sleep data through polysomnography and actigraphy[22, 23] and future research could explore how the PSQI-2 correlates with these objective measures. Second, the cross-sectional nature of our analysis precludes assessment of test-retest reliability, which would strengthen the validation of the PSQI-2. Third, while the PSQI-2 showed good performance in this multi-ethnic cohort, its performance in other populations should be verified, as sleep perceptions and reporting may vary across different cultural and clinical contexts. Furthermore, the PSQI-2 evaluated in this study is an adaptation derived from the full PSQI rather than an independently administered instrument, which may influence item interpretation. Nevertheless, this also represents a strength, as sleep duration and subjective sleep quality are commonly assessed across epidemiological studies, allowing the PSQI-2 framework to be tested and replicated in diverse datasets. This flexibility also opens opportunities for future research to evaluate alternative PSQI-2 adaptations using the same core items across different study designs and populations.

Despite these limitations, this study has notable strengths, including the large, well-characterized multi-ethnic cohort, the comprehensive psychometric evaluation using multiple complementary methods, and the consistency of findings across key demographic subgroups. The integration of actigraphy data for certain components of the full PSQI adaptation also strengthens the original measure’s objectivity against which the PSQI-2 was validated. Collectively, these results support the PSQI-2 as a valid, efficient, and scalable measure of sleep quality, particularly well-suited for epidemiological studies and clinical contexts where the full PSQI may be impractical.

## Conclusion

The PSQI-2 demonstrates strong validity as an abbreviated alternative to the full PSQI in a multi-ethnic cohort, effectively identifying poor sleep quality and maintaining consistent associations with key demographic, clinical, and sleep-related factors. Its brevity and favorable psychometric properties make it suitable for large-scale studies and clinical screening where the full PSQI may be impractical. Future research should explore the longitudinal performance of the PSQI-2, its responsiveness to interventions, and its validity in specific clinical populations. Additionally, comparison with objective sleep measures could further illuminate what aspects of sleep quality are captured by this abbreviated instrument. As sleep health continues to be recognized as essential to overall well-being, efficient and valid assessment tools like the PSQI-2 will play an increasingly important role in both research and clinical practice.

## Figures and Tables

**Table 1:** Prevalence and 95% confidence intervals of sociodemographic, health, and sleep-related characteristics according to sleep quality measures (MESA, n = 2,237)

Variable	Category	Total % (95% CI)	PSQI > 5 % (95% CI)	PSQI-2 > 1 % (95% CI)
**Sociodemographic characteristics**				
**Sex**	Female	53.6 (49.8;57.2)	(4687;58.8)	54.4 (50.1;58.7)
	Male	46.4 (42.8;50.2)	47.2 (41.2;53.3)	45.6 (41.4;49.9)
**Age group**	54;64 years	36.2 (31.6;41.1.0)	(315B;39.3)	39.4 (35.3;43.7)
	65;74 years	30.8 (28.9;32.7)	30.7 (27.7;33.8)	30.2 (28.1;32.5)
	75 + years	33.0 (29.5;36.8)	33.9 (30.4;37.5)	30.4 (26.8;34.3)
**Race/ethnicity**	White	37.1 (20.4;57.6)	34.9 (19.8;53.8)	36.5 (20.5;56.1.0)
	Black	27.5 (11.2;53.5)	29.5 (12.5;55.1.0)	28.7 (11.2;56.2)
	Hispanic	23.5 (6.7;56.7)	24.8 (7.1;58.7)	23.5 (6.7;56.9)
	Chinese-American	11.9 (2.2;45.0)	10.8 (2.0;42.1.0)	11.4 (2.0;45.0)
**Currently working**	No	57.3 (55.3;59.4)	59.0 (56.4;61.5)	55.4 (52.9;58)
	Yes	42.7 (40.6;44.7)	41.0 (38.5;43.6)	44.6 (42;47.1.0)
**Work shift**	Daytime	29.9 (28.1;31.9)	27.4 (25.1;29.7)	31.1 (28.8;33.5)
	Irregular	7.3 (6.3;8.5)	7.6 (6.3;9.1.0)	7.7 (6.5;9.2)
	Nighttime/rotating shifts	5.4 (4.5;6.4)	6.0 (4.9;7.4)	5.7 (4.6;7)
	Does not work	57.3 (55.3;59.4)	59 (56.4;61.5)	55.4 (52.9;58)
**Behavioral and health characteristics**				
Current smoker	No	93.3 (92.2;94.3)	92.7 (91.3;93.9)	92.8 (91.4;94)
	Yes	6.7 (5.7;7.8)	7.3 (6.1;8.7)	7.2 (6;8.6)
Smoking status	Former smoker	38.4 (36.4;40.4)	39.7 (37.2;42.2)	39.8 (37.3;42.3)
	Current smoker	6.7 (5.7;7.8)	7.3 (6.1;8.7)	7.2 (6;8.6)
	Never smoked	54.3 (52.3;56.4)	52.6 (50;55.1.0)	52.5 (50;55.1.0)
	Does not know	0.5 (0.3;1;0)	0.5 (0.2;1.0)	0.5 (0.2;1.0)
BMI category	Normal	27.3 (18.3;38.7)	24.2 (15.9;35.0)	25.6 (17.3;36.2)
	Overweight	37.2 (34.9;39.5)	37.6 (34.0;41.4)	37.4 (34.0;41.1.0)
	Obese	35.5 (25.3;47.2)	38.2 (27.6;50.1.0)	36.9 (26.0;49.4)
Regular naps	No	40.1 (38;42.2)	37.4 (34.9;39.9)	40.0 (37.5;42.6)
	Yes	59.9 (57.8;62)	62.6 (60.1;65.1.0)	60.0 (57.4;62.5)
Uses CPAP/BiPAP	No	94.2 (93.1;95.1.0)	27.4 (25.1;29.7)	93.4 (92;94.5)
	Yes	5.8 (4.9;6.9)	7.6 (6.3;9.1.0)	6.6 (5.5;8)
**Sleep-related conditions and components**				
Clinically significant insomnia	No	64.3 (62.3;66.3)	48.7 (46.1;51.3)	52.1 (49.6;54.7)
	Yes	35.7 (33.7;37.7)	51.3 (48.7;53.9)	47.9 (45.3;50.4)
Diagnosed sleep apnea	No	91.0 (89.7;92.1.0)	90.0 (88.4;91.5)	89.9 (88.3;91.3)
	Yes	9.0 (7.9;10.3)	10.0 (8.5;11.6)	10.1 (8.7;11.7)
Diagnosed insomnia	No	93.4 (92.3;94.4)	90.6 (89;92)	91.2 (89.7;92.6)
	Yes	6.6 (5.6;7.7)	9.4 (8;11.0)	8.8 (7.4;10.3)
Diagnosed restless legs syndrome	No	95.3 (94.4;96.1.0)	94.2 (92.9;95.3)	94.7 (93.4;95.7)
	Yes	4.7 (3.9;5.6)	5.8 (4.7;7.1.0)	5.3 (4.3;6.6)
Excessive daytime sleepiness	No	86.2 (84.7;87.6)	82.5 (80.4;84.4)	94.7 (93.4;95.7)
	Yes	13.8 (12.4;15.3)	17.5 (15.6;19.6)	5.3 (4.3;6.6)
Chronotype	Morning	41.6 (35.5;47.9)	40.0 (33.5;46.7)	40.7 (35.0;46.7)
	Intermediate	51.1 (45.3;56.8)	51.2 (44.9;57.5)	51.1 (45.3;57.0)
	Evening	7.4 (6.3;8.6)	8.9 (8.1;9.7)	8.1 (7.4;8.9)

Prevalence and 95% confidence intervals were estimated from the Multi-Ethnic Study of Atherosclerosis (MESA) Sleep Ancillary Study. Poor sleep quality was defined as PSQI ≥ 10, and reduced sleep quality by PSQI-2 as scores ≥ 3. WHIIRS: Women’s Health Initiative Insomnia Rating Scale; PSG: polysomnography.

**Table 2:** Associations between participant characteristics and poor sleep quality according to PSQI (> 5) and PSQI-2 (> 2) thresholds (MESA Sleep Study)

Variable	Category	OR (PSQI > 5) (95% CI)	OR (PSQI-2>1) (95% CI)	CI overlap
**Sociodemographic characteristics**				
Sex	Male (ref: Female)	1.10 (0.92;1.31.0)	0.90 (0.76;1.08)	*
Race/Ethnicity	Chinese-American (ref: White)	0.92 (0.69;1.22)	0.93 (0.70;1.24)	*
	Black, African-American	1.47 (1.18;1.84)	1.20 (0.96;1.50)	*
	Hispanic	1.40 (1.11;1.76)	1.05 (0.83;1.33)	*
Age group	65;74 years (ref: 54;64)	1.05 (0.85;1.30)	0.72 (0.58;0.90)	*
	75 + years	1.15 (0.93;1.41.0)	0.60 (0.48;0.74)	+
**Health and behavioral characteristics**				
BMI category	Overweight (ref: Normal)	1.42 (1.15;1.76)	1.22 (0.98;1.52)	*
	Obese	1.73 (1.38;2.15)	1.36 (1.09;1.70)	*
Current smoker	Yes	1.32 (0.91;1.90)	1.28 (0.89;1.85)	*
Work shift	Night/rotating (ref: Daytime)	1.84 (1.19;2.84)	1.06 (0.69;1.63)	*
	Irregular	1.39 (0.97;2.00)	1.05 (0.72;1.53)	*
Diagnosed sleep apnea	Yes	1.44 (1.04;1.99)	1.55 (1.11;2.16)	*
Diagnosed insomnia	Yes	7.15 (3.84;13.30)	4.07 (2.44;6.81.0)	*
Diagnosed restless legs syndrome	Yes	2.30 (1.40;3.79)	1.60 (1.01;2.53)	*
CPAP/BiPAP use	Yes	1.40 (0.95;2.08)	1.62 (1.07;2.45)	*
Regular naps	Yes	1.39 (1.16;1.66)	1.01 (0.84;1.21.0)	*
**Sleep-related scales and components**				
Insomnia severity (WHIIRS)	Moderate (ref: None)	13.04 (9.55;17.81.0)	6.54 (5.04;8.49)	
	Severe	26.42 (11.61;60.12)	10.57 (5.81;19.20)	*
Sleepiness severity (Epworth)	Mild;moderate (ref: Normal)	2.82 (2.02;3.94)	1.75 (1.29;2.38)	*
	Excessive	3.77 (1.98;7.18)	2.33 (1.32;4.11.0)	*
Excessive daytime sleepiness	Yes	2.86 (2.10;3.90)	1.80 (1.36;2.39)	*
Chronotype	Intermediate (ref: Morning)	1.13 (0.94;1.35)	1.07 (0.89;1.28)	*
	Evening	2.18 (1.47;3.24)	1.49 (1.02;2.16)	*

**Note:** Logistic regression models were estimated separately for PSQI (> 5) and PSQI-2 (> 2) as dependent variables. OR = Odds Ratio; CI = Confidence Interval; CI overlap = * indicates overlapping 95% CIs between models; + indicates non-overlapping intervals.

**Table 3:** Continuous accuracy metrics for PSQI-2 model against the full PSQI.

Model	Estimate	95%CI	
**Brier Score**	0.185	0.006	0.188
**MAE**	0.371	0.019	0.377
**RMSE**	0.430	0.075	0.434
**IDI**	0.187	0.002	0.332

Performance of PSQI-2 in predicting the continuous probability score of the full PSQI. The Brier Score measures overall prediction. Mean Absolute Error (MAE) and Root Mean Square Error (RMSE) measure the average magnitude of prediction errors. The Integrated Discrimination Improvement (IDI) approximates the difference in discrimination performance between models, calculated as the mean difference in predicted probabilities between PSQI-2 and the full PSQI.

**Table 4:** Internal validation of model-specific risk thresholds using bootstrap resampling (n = 1,000 repetitions).

Cutoff PSQI	Cutoff PSQI-2	Original Youden	Bootstrap mean	Bias	95% CI (BC)
>5	>1	0.399	0.442	0.042	0.341; 0.452
	>2	0.315	0.330	0.015	0.266; 0.361
	>3	0.104	0.119	0.015	0.074; 0.135

## Data Availability

The datasets analyzed for this study were provided by the National Sleep Research Resource (Sleep Data, https:/sleepdata.org). The MESA data are publicly available for researchers upon request. Access to the data requires approval of a data use agreement and compliance with the terms and conditions set by the MESA Study and the Sleep Data platform.

## References

[1] Philippens N, Janssen E, Kremers S, Crutzen R. Determinants of natural adult sleep: An umbrella review. PLoS ONE. 2022;17:1–30. 10.1371/journal.pone.0277323. PMC9639822 36342936

[2] Li J, Cao D, Huang Y, Chen Z, Wang R, Dong Q, Sleep duration and health outcomes: an umbrella review. Sleep Breath. 2022;26:1479–501. 10.1007/s11325-021-02458-1. 34435311

[3] Buysse DJ, Reynolds CF, Monk TH, Berman SR, Kupfer DJ. The Pittsburgh sleep quality index: A new instrument for psychiatric practice and research. Psychiatry Res. 1989;28:193–213. 10.1016/0165-1781(89)90047-4. 2748771

[4] Carpenter JS, Andrykowski MA. Psychometric evaluation of the Pittsburgh Sleep Quality Index. J Psychosom Res. 1998;45:5–13. 10.1016/S0022-3999(97)00298-5. 9720850

[5] Beck SL, Schwartz AL, Towsley G, Dudley W, Barsevick A. Psychometric evaluation of the Pittsburgh Sleep Quality Index in cancer patients. J Pain Symptom Manage. 2004;27:140–8. 10.1016/J.JPAINSYMMAN.2003.12.002. 15157038

[6] Liu X. Reliability and validity of the Pittsburgh Sleep Quality Index. Chin J Psychiatry. 1996;29:103.

[7] Wang L, Wu YX, Lin YQ, Wang L, Zeng ZN, Xie XL, Reliability and validity of the Pittsburgh Sleep Quality Index among frontline COVID-19 health care workers using classical test theory and item response theory. J Clin Sleep Med. 2022;18:541–51. 10.5664/JCSM.9658. 34534069 PMC8805004

[8] Egleston BL, Miller SM, Meropol NJ. The impact of misclassification due to survey response fatigue on estimation and identifiability of treatment effects. Stat Med. 2011;30:3560–72. 10.1002/sim.4377. 21953305 PMC3552436

[9] Ghafourifard M. Survey Fatigue in Questionnaire Based Research: The Issues and Solutions. J Caring Sci. 2024;13:214–5. 10.34172/jcs.33287. 39974826 PMC11833437

[10] de Menezes-Júnior LAA, Carraro JCC, Machado-Coelho GLL, Meireles AL. The Pittsburgh sleep quality index-2 (PSQI-2): the validity of a two-item sleep quality screener in Brazilian adults. Sleep Breath. 2025;29:238. 10.1007/s11325-025-03408-x. 40637962

[11] de Menezes-Júnior LAA. Psychometric properties of the two-item Pittsburgh Sleep Quality Index (PSQI-2) in a cohort of community-dwelling older men 2025. 10.21203/rs.3.rs-7526287/v1 PMC12972079 41803458

[12] Nishiyama T, Mizuno T, Kojima M, Suzuki S, Kitajima T, Ando KB, Criterion validity of the Pittsburgh Sleep Quality Index and Epworth Sleepiness Scale for the diagnosis of sleep disorders. Sleep Med. 2014;15:422–9. 10.1016/j.sleep.2013.12.015. 24657203

[13] Blaha MJ, DeFilippis AP. Multi-Ethnic Study of Atherosclerosis (MESA). J Am Coll Cardiol. 2021;77:3195–216. 10.1016/j.jacc.2021.05.006. 34167645 PMC8091185

[14] Ogilvie RP, Redline S, Bertoni AG, Chen X, Ouyang P, Szklo M, Actigraphy Measured Sleep Indices and Adiposity: The Multi-Ethnic Study of Atherosclerosis (MESA). Sleep. 2016;39:1701–8. 10.5665/sleep.6096. 27306270 PMC4989259

[15] Zhang G-Q, Cui L, Mueller R, Tao S, Kim M, Rueschman M, The National Sleep Research Resource: towards a sleep data commons. J Am Med Inform Assoc. 2018;25:1351–8. 10.1093/jamia/ocy064. 29860441 PMC6188513

[16] Egan KJ, Knutson KL, Pereira AC, von Schantz M. The role of race and ethnicity in sleep, circadian rhythms and cardiovascular health Short title: Sleep and cardiovascular health across ethnicities. Sleep Med Rev. 2017;33:70. 10.1016/J.SMRV.2016.05.004. 27908540 PMC6450543

[17] Carpi M. The Pittsburgh Sleep Quality Index: a brief review. Occup Med (Chic Ill). 2025;75:14–5. 10.1093/occmed/kqae121. PMC11973415 40190123

[18] Helles M, Fletcher R, Münch M, Gibson R. Examining the structure validity of the Pittsburgh Sleep Quality Index among female workers during New Zealand’s initial COVID-19 lockdown. Sleep Biol Rhythms. 2024;22:217–25. 10.1007/s41105-023-00509-6. 38524163 PMC10959842

[19] Zhong Q-Y, Gelaye B, Sánchez SE, Williams MA. Psychometric Properties of the Pittsburgh Sleep Quality Index (PSQI) in a Cohort of Peruvian Pregnant Women. J Clin Sleep Med. 2015;11:869–77. 10.5664/jcsm.4936. 25845902 PMC4513264

[20] Yang D, Li Y, Jia J, Li H, Wang R, Zhu J, Construction and validation of a predictive model for sleep disorders among pregnant women. BMC Pregnancy Childbirth. 2025;25:242. 10.1186/s12884-025-07197-9. 40050805 PMC11887067

[21] Mariman A, Vogelaers D, Hanoulle I, Delesie L, Tobback E, Pevernagie D. Validation of the three-factor model of the PSQI in a large sample of chronic fatigue syndrome (CFS) patients. J Psychosom Res. 2012;72:111–3. 10.1016/j.jpsychores.2011.11.004. 22281451

[22] Chen X, Wang R, Zee P, Lutsey PL, Javaheri S, Alcántara C, Racial/Ethnic Differences in Sleep Disturbances: The Multi-Ethnic Study of Atherosclerosis (MESA). Sleep. 2015. 10.5665/sleep.4732. PMC4434554 25409106

[23] Dean DA, Wang R, Jacobs DR, Duprez D, Punjabi NM, Zee PC, A Systematic Assessment of the Association of Polysomnographic Indices with Blood Pressure: The Multi-Ethnic Study of Atherosclerosis (MESA). Sleep. 2015;38:587–96. 10.5665/sleep.4576. 25348124 PMC4355898

[24] Schiza SE, Randerath W, Drummond M. Screening with limited sleep tests to increase pre-test probability. ERS Handbook of Respiratory Sleep Medicine. European Respiratory Society; 2023. 10.1183/9781849841641.009322.

[25] Luyster FS, Choi J, Yeh C-H, Imes CC, Johansson AEE, Chasens ER. Screening and evaluation tools for sleep disorders in older adults. Appl Nurs Res. 2015;28:334–40. 10.1016/j.apnr.2014.12.007. 26608435 PMC4661454

[26] Bild DE. Multi-Ethnic Study of Atherosclerosis: Objectives and Design. Am J Epidemiol. 2002;156:871–81. 10.1093/aje/kwf113. 12397006

